# Achieving successful community engagement: a rapid realist review

**DOI:** 10.1186/s12913-018-3090-1

**Published:** 2018-04-13

**Authors:** E. De Weger, N. Van Vooren, K. G. Luijkx, C. A. Baan, H. W. Drewes

**Affiliations:** 1 Department of Quality of Care and Health Economics, Centre for Nutrition, Prevent and Health Services, National Institute for Health and the Environment (RIVM), P.O. Box 1, 3720 BA Bilthoven, The Netherlands; 2 Tilburg University, Tranzo, Tilburg School of Social and Behavioural Sciences, PO Box 90153, 5000 LE Tilburg, The Netherlands

**Keywords:** Community engagement, Citizen engagement, Community participation, Healthcare, Rapid realist review, Realist evaluation

## Abstract

**Background:**

Community engagement is increasingly seen as crucial to achieving high quality, efficient and collaborative care. However, organisations are still searching for the best and most effective ways to engage citizens in the shaping of health and care services. This review highlights the barriers and enablers for engaging communities in the planning, designing, governing, and/or delivering of health and care services on the macro or meso level. It provides policymakers and professionals with evidence-based guiding principles to implement their own effective community engagement (CE) strategies.

**Methods:**

A Rapid Realist Review was conducted to investigate how interventions interact with contexts and mechanisms to influence the effectiveness of CE. A local reference panel, consisting of health and care professionals and experts, assisted in the development of the research questions and search strategy. The panel’s input helped to refine the review’s findings. A systematic search of the peer-reviewed literature was conducted.

**Results:**

Eight action-oriented guiding principles were identified:

- Ensure staff provide supportive and facilitative leadership to citizens based on transparency;
- foster a safe and trusting environment enabling citizens to provide input;
- ensure citizens’ early involvement;
- share decision-making and governance control with citizens;
- acknowledge and address citizens’ experiences of power imbalances between citizens and professionals;
- invest in citizens who feel they lack the skills and confidence to engage;
- create quick and tangible wins;
- take into account both citizens’ and organisations’ motivations.

**Conclusions:**

An especially important thread throughout the CE literature is the influence of power imbalances and organisations’ willingness, or not, to address such imbalances. The literature suggests that ‘meaningful participation’ of citizens can only be achieved if organisational processes are adapted to ensure that they are inclusive, accessible and supportive of citizens.

**Electronic supplementary material:**

The online version of this article (10.1186/s12913-018-3090-1) contains supplementary material, which is available to authorized users.

## Background

Ageing populations with increasingly complex health and care needs, growing health inequalities, and the challenging financial climates in OECD countries, have long emphasised the need for the provision of better and more efficient care [1]. In an effort to tackle such problems, a diverse range of organisations, including healthcare providers, insurance companies, municipalities and patient representatives are collaborating to implement new models of care [2–4]). Community engagement (CE) is increasingly seen as a key component of such new collaborative models of care. Communities often have a more holistic view of health and wellbeing, thus enabling organisations to look beyond their own interests and traditional remits [5]. The assumption is that involving communities can act as a lever for change to bring a wider range of services together even including, schools and local businesses, which would then be more tailored to the needs of the communities themselves. Many suggest that such tailored and integrated services would ultimately lead to improved community health [6, 7]. Others also believe that as citizens become more engaged and empowered to shape their local services, the management of their own health and wellbeing would also improve [8]. Many health and care organisations in the Netherlands have started implementing new CE interventions; however, there is limited insight regarding the best ways to implement successful CE initiatives.

Previous studies have evaluated different types of CE interventions that have been implemented with the aim of improving local health and care services or neighbourhoods’ healthy living infrastructure [9–12]. Earlier literature reviews have focused on how CE interventions affect populations’ health and social outcomes [8, 13] or organisational structures and processes [14, 15]. Each of these studies has shed some light on the problems that prevent CE interventions from reaching ‘meaningful’ citizen participation. For example, earlier studies have shown how power imbalances and the inaccessibility of organisational structures and processes experienced by citizens can prevent CE interventions from producing the intended outcomes and can instead lead to mistrust between citizens and professionals [9, 16–18]). However, while these earlier studies have been insightful, they do not provide professionals with the information they need to successfully implement CE interventions in their own contexts. This is partly because previous studies have provided limited insight into the ways in which the different contextual factors (e.g. existing service fragmentation) and underlying mechanisms (e.g. staff’s support and facilitation making citizens feel valued) influence CE intervention outcomes (e.g. levels of community trust).

To start providing such information, this rapid realist review (RRR) sets out eight guiding principles for ‘meaningful’ participation. The principles are based on a review of the peer-reviewed literature and are underpinned by an investigation of which CE interventions work, for whom, how, to what extent and in which contexts. The principles, along with the contextual factors and the mechanisms that influence the outcomes of CE interventions are useful for policymakers and professionals to explore when struggling with the implementation of their own CE intervention. The review specifically investigated the application of CE in health and social care, focusing on the macro and meso levels of CE, e.g. developing policies, designing, implementing and delivering health and care services, setting service and policy priorities. The review addressed the following research questions:

1. What are the action-oriented guiding principles by which community engagement interventions can be implemented successfully?
2. What are the mechanisms by which these principles operate? What are the contextual factors influencing the principles?
3. What impact do the interactions between contextual factors and mechanisms have on CE intervention outcomes?

## Methods

This review applied the rapid realist review (RRR) methodology. The realist methodology aims to highlight the impact that interactions between the contextual factors and the mechanisms have on intervention outcomes [19]. RRRs aim to provide a similar knowledge synthesis as traditional systematic realist reviews, but within a considerably shorter timeframe to ensure the relevance and applicability of results for the stakeholders [20–23].

The review was undertaken in consultation with a local reference panel. As this RRR represents the first stage of a four-year mixed methods multiple case study evaluating six community engagement interventions in the Netherlands, the local reference panel consisted of the six CE interventions’ stakeholders, including professionals, citizens and citizen representatives who will be further developing and implementing the interventions. The panel also included experts in the fields of health inequalities, citizen participation, and public health, to ensure the review addressed relevant gaps in the literature. The review followed five iterative stages, which have been applied and described by others previously [21–23]:

- Developing and refining research questions
- Searching and retrieving information
- Screening and appraising information
- Synthesising information
- Interpreting information

Because there are such wide-ranging definitions and interpretations of CE, an important first step was to find one clear definition that the authors could then apply throughout each stage of the review. Based on a preliminary search of the literature and early consultations with the panel, the authors chose the following definition of community engagement:

*‘Involving communities in decision-making and in the planning, design, governance and/or delivery of services. Community engagement activities can take many forms including service-user networks, healthcare forums, volunteering or interventions delivered by trained peers’* ([24], p. xiii).

The authors engaged with the stakeholders of the six interventions at the start of the review to ensure their key areas of interest were covered in the review, and also consulted with the other experts in the local reference panel to confirm that the review addressed relevant gaps in the literature.

In consultation with the library scientist at the National Institute for Public Health and the Environment (RIVM), and based on the chosen definition and the preliminary search of the literature, the review search terms and search strings were agreed (See Additional file 1) and applied in the electronic databases, Embase and Scopus. These two databases were chosen as they were deemed by the library scientist to be the most relevant to the review’s subject area. Furthermore, Embase and Scopus are two of the largest international databases with a focus on health and social research and include trade journals as well. Upon reviewing the results of these two databases, the authors felt that enough rich data had been obtained and so did not search any other databases in order to speed up the process to ensure the stakeholders received the relevant information on time and in line with their CE intervention implementation schedules.

The draft inclusion and exclusion criteria were developed based on the preliminary search and were tested by two reviewers (EdW and NvV). Based on this test, the reviewers decided to expand the original criteria to ensure closer alignment with the review’s scope and the chosen CE definition. The reviewers screened the papers in two stages. During the first stage, papers’ titles and abstracts were screened, for example, based on whether the CE interventions described involved citizens or communities in the decision-making, planning, design, governance or delivery of health and care services or policies. EdW and NvV applied these criteria to the titles and abstracts and rated papers: (a) ‘red’, if papers did not follow the agreed definition of CE and/or if topics fell clearly outside the scope; (b) ‘amber’ if this was unclear; or (c) ‘green’, if the papers clearly applied the same definition and discussed topics within the scope. Initially, EdW and NvV both screened the same 100 papers to ensure standardisation of the screening process. After this, the reviewers each reviewed a different stack of papers to speed up the screening process. EdW and NvV crosschecked and discussed the papers rated ‘amber’ or ‘green’ to ensure consistency in their approach. Additionally, HD sampled 40 papers—20 papers which NvV and EdW had both screened, 10 papers which EdW had screened and 10 papers which NvV had screened—to ensure EdW’s and NvV’s screening was rigorous, consistent and standardised. Papers rated ‘red’ did not continue to the second, full-text, screening stage (Table 1).

**Table 1:** Title and abstract criteria

Inclusion criteria	
English peer-reviewed literature	
Paper discussing CE interventions involving citizens or communities in the decision-making, planning, designing, governance, and/or delivery of health or care services and/or policies	
Papers set within OECD country	
Exclusion criteria	
Unpublished literature, papers which were difficult to obtain	
Papers discussing CE interventions NOT involving citizens or communities in the decision-making, planning, designing, governance, or delivery of health and care services, or policies	
Papers discussing CE interventions which only involved citizens or communities in health-research	
Papers not set within OECD countries	
Papers not set within a health or wellbeing context	
Papers published before the year 2007	

During the second screening stage, EdW and NvV assessed the full text of those papers that had been rated ‘green’ or ‘amber’ for methodological rigour using the *Mixed Methods Appraisal Tool (MMAT)* [25] and for relevance. Relevance was assessed by asking questions like whether CE was the paper’s main subject area and whether the CE interventions described operated on Rowe & Frewer’s [26] ‘Public Participation’ level. In line with the O’Mara-Eves et al. [24] definition, the authors used Rowe & Frewer’s [26] classification of public participation to assess whether the interventions described in the literature operated on the ‘public participation level’ whereby citizens are not merely receiving information from organisations (public communication level), or merely providing information to organisations (public consultation), but are actively engaged in dialogue with organisations (Table 2) [26].

**Table 2:** Full text exclusion and relevance criteria

Exclusion	
Does the paper focus on CE as the main subject area or as an important aspect of a wider programme? Papers only tangentially describing CE were excluded	
Does the CE intervention, described involve citizens or communities on the macro or meso-level? Papers concerned only with micro-level CE interventions were excluded (e.g. individual social participation)	
Does the paper focus on CE as the main subject area or as an important aspect of a wider programme? Papers only tangentially describing CE were excluded	
Does the CE intervention operate on Rowe & Frewer [26] ‘Public Participation’ level? Papers concerned with interventions solely based on the ‘Public Communication’ or ‘Public Consultation’ levels were excluded	
Relevance	
Does the paper describe contextual details? OR	
Does the paper describe mechanisms? OR	
Does the paper describe CE strategies, processes implemented? OR	
Does the paper describe CE models, theories applied? OR	
Does the paper describe the engagement of disadvantaged/vulnerable groups? OR	
Does the paper discuss health and wellbeing outcomes of CE intervention? OR	
Does the paper describe CE as way of developing intersectoral approaches/new models of collaborative care?	

Data extractions were conducted on the final set of selected papers using an extraction template *(available upon request)*. The template was used to extract data regarding the interventions’ strategies, activities and resources, and the context, mechanisms and outcomes directly stipulated in the papers. To aid the reviewers during the extraction process and to ensure consistency and transparency, the authors specified CE-oriented definitions of important realist concepts. The realist methodology is still developing and as such, realist evaluators continue to unpack and operationalise terms like ‘context’, ‘mechanisms’, and ‘interventions’ and how these interrelate [27]. The following CE-oriented definitions of the realist concepts were applied:

- *Intervention*: refers to interventions’ implemented activities, strategies and resources [27] e.g., citizen advisory panel meetings, neighbourhood clean-up activities, or citizen learning opportunities.
- *Mechanism*: the concept of ‘mechanism’ does not refer to the intentional resources offered or strategies implemented within an intervention. Rather, it refers to what ‘triggers’ participants to want to participate, or not, in an intervention. Mechanisms usually pertain to cognitive, emotional or behavioural responses to intervention resources and strategies [28], e.g., citizens feeling more empowered due to learning opportunities.
- *Context*: pertains to the backdrop of an intervention. Context includes the pre-existing organisational structures, the cultural norms and history of the community, the nature and scope of pre-existing networks, and geographic location effects [28, 29], e.g., pre-existing levels of trust between communities and organisations or previous experience of CE interventions.
- *Outcome:* refers to intended or unexpected intervention outcomes [28] e.g. sustainability, quality integration of services (macro); citizens’ level of involvement in health and care services (meso); citizens’ health and wellbeing outcomes (micro).

Using completed extractions, EdW and NvV created Context-Mechanism-Outcome configurations (CMOs) in order to understand and explain why CE interventions work, or not, and to generate the action-oriented guiding principles. For this review, the authors only created CMOs if those contexts, mechanisms and outcomes were explicitly correlated in the papers themselves to avoid conjecture. After drafting the configurations, the mechanisms of the CMOs were first clustered per type of CE intervention in order to ensure that the eventual principles were underpinned by mechanisms found across the range of CE interventions and thus across different contexts—i.e. (a) citizens involved in health and care organisations or neighbourhood panels, forums, boards, steering groups, planning and decision-making committees; (b) community-wide volunteering and community group activities in health and care related subjects; and (c) peer delivery. After this initial round of clustering, NvV and EdW searched for keywords in those mechanisms and then thematically clustered the mechanisms according to those keywords—independent of the types of intervention—thus generating the guiding principles. As papers were able to contribute to multiple principles, EdW and NvV also checked that each principle was based on mechanisms from several different papers to ensure the principles were transferable across different interventions and contexts. The final draft of the clustered mechanisms was shared with the other authors to confirm the mechanism themes and to refine the principles. The mechanisms of the CMOs were chosen as the basis for generating the principles, because the question of what makes citizens want to participate or not, are central to the CE literature and to the local reference panel. This question is inherently related to the concept of ‘mechanisms’—what ‘turns on in the minds of program participants and stakeholders that make them want to participate or invest in programs’ [28].

Finally, the authors held a workshop in order to present the review’s findings, including the final draft of the principles, to the local reference panel. During the workshop, the panel discussed the applicability of the principles within their local contexts and whether they are experiencing similar issues in the development of their own CE interventions. Confirming that the final draft of principles and their corresponding mechanisms had face validity, the workshop provided rich anecdotal evidence, thus further refining and finalising the principles discussed below.

## Results

**Fig. 1:**
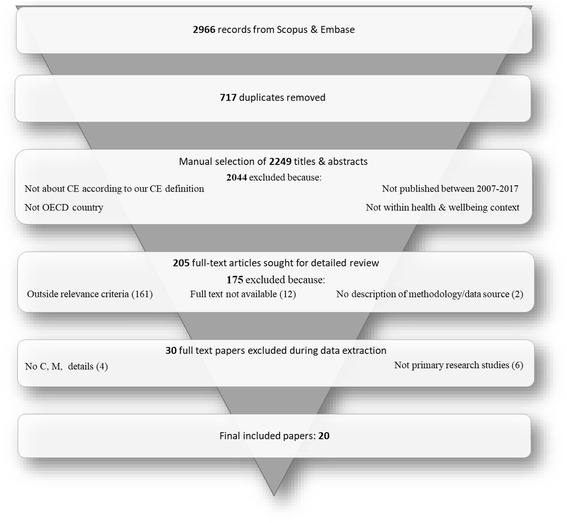
Flowchart of document inclusion and exclusion process

After the removal of duplicates, the search resulted in 2249 potentially relevant papers (see Fig. 1). After the first title and abstract screening stage, 205 papers were selected to continue to the second full text screening stage. After applying the full-text inclusion and exclusion criteria and removing a further four papers as they contained no information on contexts or mechanisms and excluding six literature reviews to ensure this review’s findings were based on primary data, a total of 20 papers were used for data extraction.

The majority of papers focused on CE interventions involving citizens in healthcare organisations’ or neighbourhood panels, forums, boards, steering groups, or planning and decision-making committees [9, 17, 18, 30–40]. For example, [32] study described the Australian District Aboriginal Health Action Groups (DAHAGs), which included both Aboriginal community members and healthcare professionals who together, identified local solutions to important Aboriginal health problems [32]. Five papers investigated CE interventions involving community group activities or community wide volunteering ([11, 12, 31, 41]; Schoch-Spana). For example, Hamamoto et al. [11] described how community volunteering and actions groups, together with the local Community Centre, developed and implemented a new healthy living infrastructure in the local neighbourhood. Only two papers described peer delivery interventions [10, 16]. For example, De Freitas & Martin’s [16] study showed how Cape Verdean migrant mental health service-users were empowered and actively engaged in supporting and recruiting other service-users (Table 3).

**Table 3:** Summary of CE interventions implemented

Paper	Care setting	Type of participation & main strategies used	Overall outcomes
Carlisle (2010) [9]; ethnographic study	Public health	*Social Inclusion Partnership set up to tackle social exclusion and health inequalities within deprived local neighbourhoods* - SIP included representatives from the local authority, primary care, benefits agency and the police, six positions were reserved for community representatives. Twelve residents were recruited to form a community sub-group to work with the SIP chair and manager to develop process for selecting community representatives. - SIP allocated funds and resources to projects and services promoting the health of local population	- Difficulties securing community representation on SIP, especially young residents - Priorities of professionals and residents on SIP were not aligned - Due to enduring disagreements the leader of the community representatives and the SIP chair resigned
Chan & Benecki (2013) [30]; Qualitative case study	Hospital	*Citizens’ Advisory Panel (CAP) developed efficiency and operational recommendations for Hospital Board* - External consultancy with experience in CE strategies assembled CAP and facilitated their meetings, e.g. by setting tasks like gathering community input on specific topics - Hospital board maintained two-way open communication with CAP	- Majority of respondents felt CAP was an effective way to incorporate community’s perspective in decision-making - Most CAP members said they would participate in similar CE interventions - Board approved the majority of CAP recommendations, which resulted in a balanced budget
Clark et al. (2010) [31]	Disease-specific community coalition	*Broad community-based asthma coalitions* - Health in All policies approach regarding membership of the coalition (e.g. community providers, schools, patients, parents, hospitals, charities) who all shared concerns regarding asthma prevention and care; ensured at least ¼ of core members were residents or community-based groups - Coalitions aimed to establish leadership which takes into account each members’ needs and concerns - Periodic joint meetings to enabled the coalition to discuss processes and outputs, set clear coalition scope and geographic boundaries, provided continuous feedback and provision of expert assistance if needed	- Fewer asthma symptoms reported among children and greater sense of control in managing the disease for parents - Overall participation rates were highest among community-based groups (e.g. parent or advocacy groups, faith-based groups, youth organisations) and health care providers
Crondahl & Eklund (2015) [10]; (qualitative) participatory action research	Community, health promotion	*Work Integrated Learning programme (health promotion & peer-support):* - Seven Roma residents employed and trained to work as local health promotion coordinators to empower their local Roma community. - Programme included theoretical module regarding community organising, social determinants of health and health promotion, sources of oppression and discrimination. Practical module allowed coordinators to work in their local communities thus practicing and applying the theoretical training. - Coordinators held interviews with local media to promote their activities	- Enhanced coordinators’ self-acceptance, positive sense of Roma identity and community, self-efficacy skill and sense of control - Introduced coordinators positively to non-Roma society
De Freitas & Martin (2015) [16]; qualitative case study	Community, mental health care	*Peer-support &-recruitment within community mental health advocacy programme promoting Cape Verdean migrants’ rights and access to mental healthcare*: - Service-user committee disseminated information about the project, enabled dialogue between service-users, providers and health authorities and held meetings outside organisational sphere. - Service-user peer support group enabling emotional and social support - Training sessions raising service-users’ awareness regarding causes of their disadvantage and tools to help alleviate these	- Enhanced peer-supporter and recruiter health literacy, confidence, communication skills, empowerment
Durey et al. (2016) [32]; qualitative multiple case study	Community and hospital	*Strategy and priority setting forums consisting of Aboriginal community members and healthcare professionals* - District Aboriginal Health Action Groups (DAHAGs) were located within the structure of the Department of Health in Western Australia and made recommendations to improve health service delivery for Aboriginal people, which the health services were responsible for implementing. - Aboriginal community members in the DAHAGs were nominated by their peers to sit on the DAHAGs for a two-year term. - Community members received governance training, e.g. in meeting procedures, chairing of meetings so they could chair DAHAG meetings	- Improved Aboriginal community capacity - Improved Aboriginal satisfaction with community and hospital setting - Increased levels of trust in local health services - Improved Aboriginal access to care
Hamamoto et al. (2009) [11]; qualitative case study	Community	*Community volunteering & action groups to develop new healthy living infrastructure* - Community Health Centre engaged community members in tangible projects (e.g. development of unused state park; bicycle repair & distribution programme) - Used a flexible, project-oriented and task-specific approach to enable each project to develop its own distinct set of volunteers and organisational partners. - Monthly scheduled volunteer workdays and used local media to promote activities	- Reclaimed 100 acres of new green space for active-living purposes - Community involvement in tangible projects: 60 volunteers weekly & 50 volunteers each month for community workdays
Kegler et al. (2009) [41]; mixed methods multiple case study	Community	*California Healthy Cities & Communities Program- community volunteering & action groups* - Local residents engaged in the programme through membership of community-based coalitions to develop shared vision, conduct asset-based community assessments, set priorities, develop and implement action plans. - Residents involved in each programme aspect in e.g. conducting focus groups, community mobilisation activities - Leveraged professional coordinators and volunteers that were well connected within the communities and widely promoted initiatives through local media	- Half of coalitions comprised 75% of residents in the planning phase - Most coalitions maintained at least 50% resident composition during implementation phase - Continued challenges engaging Hispanic residents
Kelaher et al. (2014) [33]; mixed-methods multiple case study	Regional governance in Aboriginal health	*Regional planning forums responsible for the planning, implementation and governance of the Aboriginal Health National Partnership Agreements* - Forums consisted of local Aboriginal community members and Aboriginal Community Controlled Health Services (ACCHSs) which were governed by board of directors elected by the community they serve, mainstream health providers. - Forums mindful to engage Aboriginal community organisations in all phases of planning and governance - Some forums privileged the views of Aboriginal organisations and community members by ensuring they were co-chaired by an ACCHS representative and the director of the regional health department branch.	- Forums provided an opportunity for engagement that was not funded previously - Forums provided opportunity for mainstream and Aboriginal organisations to work together in a more collaborative way to achieve better health
Lang et al. (2013) [12]; literature review and qualitative multiple case study	Community setting, health and social domain	*Top-down and bottom-up cooperative governance structures for citizen participation in service provision* - Case 1: the municipality invited residents to join the local initiative but retained full decision-making powers. Design of social service carried out in the traditional way - Case 2: delivery of public service planned by local representatives and citizens. Highly respected local representatives were involved in initiative from the start who engaged and involved residents - Case 3: Delivery of public service planned by local representatives with support from mayor and other local politicians. Idea was that as the initiative matured, resident participation would broaden and include a volunteer activities for the whole community	- Case 1: residents did not feel as if they had ownership of the initiative - Case 2: most residents signed up to the membership and the local council and federal state provided financial support for the cooperative. Management and decision-making reflected local community as residents held decision-making positions - New public service widely used by residents and led to a better range of fresh healthy food being available and a new central meeting point for residents
Lewis (2014) [17]; ethnographic study	Mental health (hospital and community)	*Service-user involvement in mental health services* - Service-user group involved in a local psychiatric hospital. Its main purpose was to provide information about mental health services and activities in the community and to provide service-user feedback and input. - Voluntary sector community group, which included practitioners and service-users which undertook lobbying activities in relation to mental health policy and services. - Professionals in these groups suggested they extended ‘standing open invitations to people for policy meetings and stated they aimed to better include service-users by giving them information before meetings and buddying them up to attend the meetings. Training initiatives aimed at facilitating service-users’ participation were implemented after the study	- Reinforced hierarchical and power relations which denied service-users equal status - Some service-users felt that gaining access to decision-making bodies was in and of itself an important achievement; while others felt excluded and silenced. - Ultimately, service-users formed their own groups and initiatives
Luluquisen & Pettis (2014) [34]; qualitative case study	Community	*Residents’ collaborative to improve neighbourhood conditions for healthy living* - From the onset, CE was made a priority and residents were integral to leadership and decision-making in the development and design of priorities, strategies and programmes and the collaborative steering committee - Collaborative included capacity-building training and initiatives for residents; to take on leadership positions in the collaborative, their communities and the broader policy arena - Youth Action Board to give young residents a voice and ensure a youth lens for the collaborative - If there are multiple organisational representatives from one organisation their votes count as one	- Higher percentage of free, healthy breakfast programmes in schools - Higher number of resident volunteers in improving access to healthy food - Improved resident skills and knowledge - Improved access to healthy food in neighbourhood
Montesanti et al. (2015) [35]; qualitative research with primary care professionals	Community, primary care	*Community Boards, which include members of local marginalised population, govern Community Health Centres* - Suggestions included only initiating CE interventions after sufficient information is gathered on the local population’s characteristics - Leveraging practitioners who have worked in the local community for a longer period of time - Enabling local population to decide how they want to be engaged	- Community members of marginalised communities are more comfortable providing input into the planning or decision-making of services with staff members who have spent years building relationships
Pennel et al. (2015) [36]; mixed-methods research -multiple sites	Hospital	*Non-profit hospitals mandated to conduct Community Health Needs Assessments using community participation* - One site had its own board of community members, which provided input on community health needs - Another hospital held a community summit, which was attended by 100 community stakeholders & members. Attendees were asked to prioritise top three community health issues and to develop goals & actions for each topic area - Most hospitals only sent out surveys to community	- The majority of hospitals only consulted with communities - 4% if hospitals involved communities in final priority selection of health needs. - 2% of hospitals involved the community in the selection of strategies to address health issues
Renedo & Marston (2011) [18]; ethnographic study -multiple sites	Primary care, acute care, hospital care	*Patient and public involvement in organisations that provide and commission care* - Training programme aimed at creating ‘effective’ community members to develop ‘professional skills’ - Placed service-users within specific disease-expert categories (e.g. HIV) to elicit specific service-user experiential input	- CE mostly at consultation level
Schoch-Spana et al. (2013) [42]; qualitative research – multiple sites	Public health emergency preparedness	*Health Departments engaging local communities to ensure preparedness in case of emergencies and disasters* - Recommended combining public deliberation methods with mobilising volunteers and ensuring citizens are involved in planning and decision-making sessions. - Suggested leveraging the shock communities and organisations feel after natural or man-made disaster to engage with each other	- Citizens more likely to come forward asking for information after a disaster - After disaster communities and organisations more open to community engagement to promote community preparedness
Tenbensel et al. (2008) [37]; qualitative -multiple sites	Primary care	*District Health Boards aimed to engage communities to ensure strategies reflected local populations needs and to orientate health sector towards population health* - DHBs comprised of locally elected board members and community group representatives and facilitated community input through population health needs assessments, open board and committee meetings, formal consultation processes - DHBs had to follow formal planning documents to meet central government requirements	- Particularly in DHBs with large populations, there was wide variety in level and nature of CE activities - Only 55% of respondents felt that DHB decisions were influenced by community input. Community input appeared less influential in strategic planning and more so in more specific areas of service design and delivery
Van Eijk & Steen (2016) [38]; qualitative multiple case study	Elderly care, disabled persons care	*Service-user council for elderly healthcare provider and for disabled persons* - Patients, family members, voluntary caregivers, and even neighbours can become members of the client council. For one of the councils, members are elected for a four-year period. - The council deliberated on the organisation’s management and quality of care and were responsible for representing all clients. Some organisational decisions could not be made without the council’s permission.	- Client council consistency at risk due to high member turn-over
Veronesi & Keasey (2015) [39]; qualitative multiple case study	Mental health care, acute care	*Patient and Public interventions to leverage organisational and strategic change* - Broad upfront consultation aimed at all potentially interested parties, open discussions with key stakeholders (staff, local population, patient representatives, voluntary organisations, local authority), and once decisions were taken, a feedback questionnaire submitted to the wider stakeholder base. - Strategic working groups including both professionals and patient and public representatives met regularly to discuss any relevant strategic matters to focus on innovative ways to delivery care	- Improvement in community’s overall attitude to organisational management - Reorganisation plans approved and implemented which resulted in improved clinical targets
Yoo et al. (2008) [40]; Community-based participatory research-multiple sites	Community, housing	*Resident groups in senior housing identified health issues and developed and implemented strategies and improvement plans for community empowerment* - Resident panels were set up with support of researchers and included interested residents and elected officers of pre-existing tenant organisations - Panels conducted brainstorming sessions to identify community health priorities, and continued to develop strategies and implementation plans to address those priorities - Some panels followed more formal structure with the signing of a memorandum of agreement, others incorporated Panel meetings into the tenant council meetings.	- Some panels were able to take steps to improve tangible aspects of everyday living, e.g. access to age-appropriate exercise equipment, social events like movie nights - Not all panels were able to proceed to implementation stage due to, e.g. conflicts of interests

A total of eight guiding principles was identified through the literature and enriched and triangulated by the panel’s input. Table 3 summarises the enabling contexts and mechanisms underpinning the principles that organisations can build on to ensure CE interventions are successful. It is worth noting that constraining contexts and mechanisms are largely two sides of the same coin—e.g. lack of previous positive relationships between organisations and communities *(context)* and lack of quick wins worsened residents’ feelings of hopelessness and powerlessness *(mechanism)*. The following section first describes each principle using the evidence from the literature review, including examples of individual CMO configurations underpinning the principle *(full list of individual CMO configurations available upon request)*. After each principle, the panel’s reflections will be summarised separately—the panel’s input did not change the wording of the principles and instead triangulated and enriched the literature findings (Table 4).

**Table 4:** Summary of guiding principles and corresponding supportive interventions, contextual factors and mechanisms leading to successful CE interventions

Interventions	Enabling contextual factors	Enabling mechanisms	Relevant citations
Guiding principle 1: Ensure staff provide supportive and facilitative leadership based on transparency			
Provide citizens access to all relevant resources Implement two-way communication with citizens Facilitate citizens’ understanding of key topics	Accessible points of connection between communities & local services Supportive organisational structures Unique points of connection between communities and local services	Staff’s support and facilitation makes citizens feel valued Professionals openly listening to citizens’ problems and ideas, improves professionals’ understanding of communities’ needs Transparency about limited resources can prevent communities from feeling frustrated	Chan & Benecki [30] Durey et al. [32] Tenbensel et al. [37] Yoo et al. [40]
Guiding principle 2: Foster a safe & trusting environment to enable citizens to provide input			
Invest resources in the building of trusting relationships with communities Tailor strategies to citizens’ needs and preferences Hold meetings outside organisational sphere Adjust meetings and activities to citizens’ needs (e.g. language, timetable) Citizens to (co)chair boards, steering groups Hire demographically and culturally diverse staff in order to better reflect and connect with the communities	Accessible organisational structures Community members included in governance and leadership of intervention and engaged in decision-making processes Pre-established trusting relationships with communities	Culturally safe spaces build communities’ confidence to discuss their needs Staff who create safe environments and address citizens’ supportive needs help build trust and cohesion	De Freitas & Martin [16] Durey et al. [32] Kegler et al. [41] Kelaher et al. [33] Luluquisen & Pettis [34] Montesanti et al. [35] Schoch-Spana et al. [42] Veronesi & Keasey [39]
Guiding principle 3: Ensure citizens’ early involvement			
Discuss with citizens the stage at which they want to be involved Align organisational and citizens’ health definitions and priorities Include citizens in needs assessments and identification of priorities	Financial or quality related organisational crises highlighting need for far-reaching change Pre-established collaborative relationships	Early involvement motivates and enables all stakeholders to bring about change Early involvement of some citizens can trigger others to become involved as well	Carlisle (2010) Clark et al. [31] Lang et al. [12] Tenbensel et al. [37] Veronesi & Keasey [39]
Guiding principle 4: Share decision-making and governance control with citizens			
Adjust decision-making methods by having multiple professionals from the same organisation share one vote on decision-making committees, thus levelling out the vote share Place citizens in leadership and decision-making positions Share relevant resources and tools with engaged citizens	More in-depth collaboration between partners Interventions initiated by citizens themselves Organisations willing to address power imbalances	Citizens’ willingness to join intervention depends on extent to which organisations are ready to share control Satisfaction rates of CE forums increases with number of involved citizens Increasing citizens’ input during strategic and decision-making stages is valued by citizens and helps prevent feelings of disempowerment	Carlisle (2010) [9] Clark et al. [31] Durey et al. [32] Kelaher et al. [33] Lang et al. [12] Luluquisen & Pettis [34]
Guiding principle 5: Acknowledge and address citizens’ experiences of power imbalances			
Invest in communities with low levels of readiness to build their capacity Adjust organisational approaches, structures, processes by privileging citizens Allow citizens to shape their own role	Inclusive organisational structures Equal number of citizens and professionals in leadership and decision-making positions Clear remits for professionals and citizens	Clear recognition of citizens’ valuable contributions, legitimises initiatives Equal presence of citizens on forums prevents citizens from experiencing being at the lower end of the power spectrum	Carlisle (2010) [9] Kelaher et al. [33] Lewis [17] Luluquisen & Pettis [34] Renedo & Marston [18]
Guiding principle 6: Invest in citizens who feel they lack the skills and confidence to engage			
Provide professional or leadership training, e.g. in chairing meetings, conducting support-group sessions Provide learning opportunities highlighting causes of citizens’ disadvantage and tools to alleviate these	Citizens motivated to improve their neighbourhoods and services they access	Improved awareness helps citizens to develop greater sense of control, self-confidence, skills Being involved in direct peer recruitment can lead to service-users recognising their own entitlement to participation	Crondahl & Eklund Karlsson [10] De Freitas & Martin [16] Durey et al. [32] Lang et al. [12] Renedo & Marston [18]
Guiding principle 7: Create quick and tangible wins			
Offer short-term mobilisation activities, e.g. neighbourhood clean-ups Ensure citizens’ input is actually used Use local media to share quick win stories	Pressing and visible health and socio-economic needs combined with significant community support for change	Early successes provide momentum, creates trust in CE processes and inspires other citizens to become involved Short-term concrete improvements can maintain citizens’ dedication to CE processes when problems arise	Durey et al. [32] Hamamoto et al. [11] Kegler et al. [41] Luluquisen & Pettis [34] [40])
Guiding principle 8: Take into account both citizens’ and organisations’ motivations			
Be flexible and allow citizens to focus only on those issues that interest them Use crises situations to catalyse citizen engagement Be transparent about organisational motivations and requirements Be open and receptive to citizens’ negative service-usage experiences	Pressing and visible health and socio-economic needs and significant community support for change Service-users and carers wanting to increase level of social interactions, and to upskill	Catering to citizens’ motivations helps maintain momentum Building on citizens’ emotional links to neighbourhood or services can connect citizens Crises situations can mean organisations are forced to change their traditional patterns	De Freitas & Martin [16] Hamamoto et al. [11] Lang et al. [12] Lewis [17] Pennel et al. [36] Schoch-Spana et al. [42] Van Eijk & Steen [38] Veronesi & Keasey [39]

### Guiding principle 1: Ensure staff provide supportive and facilitative leadership to citizens based on transparency

Supportive and facilitative leadership refers to organisational leadership that supports citizens in their roles and tasks without being too directive or restrictive. Such support should be based on transparency allowing both citizens and professionals to easily share information with each other. This helps to ensure that all those involved in CE interventions are clear on the expected outcomes [30, 32, 37, 40]. One of the examples from the literature involves a hospital setting up a Citizen’s Advisory Panel not just to address the hospital’s significant deficit, but also to create community support for the required service changes and to foster closer relationships with the community. From the start, the Board was transparent about the difficult financial situation and stipulated that the Panel’s role was to make far-reaching recommendations regarding the Hospital’s operations and processes in order to make the hospital more efficient [30]. The Board supported the Panel, for example, by giving and receiving presentations and by enlisting the help of an external consultancy who facilitated the Panel in developing their recommendations. While the Panel felt anxious about the magnitude and complexity of their task and their own recommendations on how best to address the hospital’s significant deficit *(context)*, the supportive yet not directive facilitation and transparency of the Board ensured that the Panel remained positive and motivated throughout the process (*mechanism*). Ultimately, the Panel members stated they would engage in such interventions again *(outcome)*. Furthermore, the Board approved the majority of the Panel’s recommendations (*outcome*), which resulted in a balanced budget (*outcome*). While some in the wider community were angry about the service cuts, the overall response of the community was positive (*outcome*) [30].

As evidenced in Table 3, CE interventions operate within a wide range of contextual factors relating to leadership. Enabling contextual factors include previous positive experiences of CE [30] and organisational structures providing separate points of connections between communities and local services (e.g. quarterly meetings, whole-area forums) [32]. Constraining contextual factors include engaging communities with pre-existing low-levels of community readiness to mobilise around a health or neighbourhood issue or citizens with deteriorating health [40]. If contextual constraints are not acknowledged, interventions will likely be met with resistance. For example, unsupportive leadership that is unable to release control to citizens living in low-income neighbourhoods, leads to those citizens feeling frustrated and disempowered [40]. However, the literature shows that CE interventions operating within constraining contextual factors do not automatically fail as long as such constraints are acknowledged and addressed within the intervention by supportive and facilitative leadership [30, 32, 40].

### Local reference panel reflections

The panel acknowledged the importance of fostering supportive leadership and offering specific points of connection between communities and their local services. The panel proposed having one consistent professional in a leadership position whom citizens can contact if they need further information or support.

#### Guiding principle 2: Foster a safe and trusting environment enabling citizens to provide input

Creating forums where citizens and professionals alike feel comfortable enough to put forward ideas is critical to CE interventions’ success. Engagement processes and activities should, therefore, be adjusted to suit citizens’ needs and organisations should take steps to reduce practical as well as cultural barriers to enable their full participation [16, 32–35, 39, 41, 42]. Examples from the literature include holding meetings and activities when convenient to citizens, taking into account citizens’ language needs (e.g. less jargon), and ensuring activities aimed at ethnic minorities are culturally sensitive [16, 34, 42]. In Schoch-Spana et al.’s [42] study the enabling organisational infrastructure *(context)* helped management to create a safe environment for the community to ask questions during deliberative meetings, which helped to build trust and cohesion *(mechanism)*. Management’s efforts, in turn, enabled staff to dedicate time to building trusting relationships with the community *(outcome)* which then meant that citizens were more likely to come forward and volunteer their own time *(outcome)*. Luluquisen & Pettis’s [34] study highlighted that it is important for organisations to consider citizens’ potentially differing needs and cater to different groups so that safe spaces can be created for those different groups (e.g. a youth only steering group, separate from adult boards). Creating a safe and trusting environment is especially important in contexts of marginalisation and racism. Often, such communities are mistrustful of local services [42], especially if past engagement efforts have failed to bring any improvements [32]. Failing to accommodate citizens’ needs would result in citizens feeling intimidated by, e.g. professional meetings [41]. Before implementing any plans, organisations will need to invest time and resources into addressing these contextual factors [16, 32, 35, 42].

### Local reference panel reflections

The panel emphasised that local neighbourhoods do not consist of one homogeneous group of citizens with the same interests and needs. This means that local citizens sometimes have opposing views and priorities and that organisations should play a mediating role by, for example, setting up safe forums where such tensions can be openly discussed.

#### Guiding principle 3: Ensure citizens’ early involvement

Citizens should be involved as early as possible, though the point of citizen involvement should be discussed with citizens. Where possible, organisations should engage citizens in the identification and prioritisation of their own healthcare needs. In doing so, organisations ensure that their priorities and definitions of health are aligned with those of the citizens they serve [9, 12, 31, 37, 39]). Veronesi & Keasey’s [39] study showed how the early involvement of citizens was critical in overcoming initial staff resistance to the proposed reorganisation of an acute hospital. While staff was resistant to any change, the local community and patient representatives welcomed the chance to improve the failing local hospital and thus became active drivers for change. [30] study is particularly interesting, as the conflict of interest did not centre on the differences of opinions between ‘the organisation’ as a whole and ‘the community’. Instead, the organisation’s upper management seemed at odds with its staff, thereby creating a unique opportunity to leverage the community’s input to make the required changes. The literature includes several examples of how failing to include citizens early on negatively affected the outcome of CE interventions [9, 12, 37]). For example, Carlisle (2010) evaluated a Panel consisting of professionals and local community members tasked with tackling the social exclusion and health inequalities experienced by deprived local communities *(context)*. However, the Panel had already been operational for over a year before any community members were able to join *(context*). Because the professionals in the Panel had already allocated funds and resources, the community members felt ‘like tokens’ on the Panel *(mechanism)* and were keen to ‘present a united front’ against the professionals *(outcome)*. This ultimately led to a tense and uncollaborative relationship *(outcome)* [9]. Organisations will struggle to involve citizens early on, if contextual power imbalances between professionals and citizens are not addressed and organisations maintain overall control of interventions’ projects and plans. Ultimately, citizens who are shut out of strategic and decision-making stages end up feeling disempowered and demotivated to continue their engagement [12]. Instead, the early involvement of citizens can build momentum and motivate others to join CE interventions [12, 31].

### Local reference panel reflections

While the panel recognised that early involvement of citizens is important, in the panel’s experience, citizens often struggle to participate if organisations have not yet worked out any concrete goals or plans, as they prefer having something tangible to discuss. They suggested organisations support citizens to turn their own ideas into workable plans and strategies.

#### Guiding principle 4: Share decision-making and governance control with citizens

Organisations should encourage citizens to take on governance and decision-making roles within CE interventions [9, 12, 31–34]). The literature includes many examples of how organisations maintain control of the management, governance and planning of CE interventions [9, 12, 31, 33]). For example, in Carlisle’s study (2010), once the citizens had joined the Panel, the professionals continued to maintain control by monopolising the meetings by ‘wading through large quantities of complex paperwork’ and the tenant council of one of the interventions evaluated by Yoo et al. [40] maintained control by, for example, cancelling meetings at the last minute. The literature also highlights examples of how organisations can share control by amending interventions’ governance and management structures and processes [12, 32–34]. For example, Durey et al.’s [32] study showed that in the context of the community’s marginalisation and mistrust of culturally inappropriate mainstream health services *(context)*, enabling Aboriginal community members to choose their own representatives on the DAHAGs was valued by community members *(mechanism)* and led to more authority being placed in the hands of the community *(outcome)*. Renedo & Marston [18] took a broader view and recommended that organisations examine the way in which professionals view and discuss citizen participation and to enable citizens to shape their own roles and identities instead. This way citizens will not have to adapt to organisations’ ‘elite systems’ and are valued for their own unique input. Such sharing of control is harder to achieve in contexts of marginalised communities with lower levels of readiness and hierarchical organisational structures, and when interventions have been developed ‘top down’. In such contexts, engaged citizens quickly feel as if professionals dismiss their views [34]. Ultimately, as Lang et al. [12] highlighted, citizens’ willingness to participate in interventions significantly depends on the extent to which organisations are willing and able to share control.

### Local reference panel reflections

The citizens and citizen representatives on the panel echoed the review’s findings and suggested organisational processes should be more tailored to citizens as they find it difficult to navigate organisational processes and structures. In their experience, citizens are often unaware of which organisations or professionals to approach with their ideas or what processes they are expected to follow.

#### Guiding principle 5: Acknowledge and address citizens’ experiences of power imbalances between citizens and professionals

Addressing power imbalances between citizens and professionals is crucial to CE interventions’ success. However, there are several factors, which contribute to citizens’ relative powerlessness [9, 17, 18, 33, 34]). Firstly, as we have seen, organisational structures and hierarchies are tipped towards professionals rather than citizens leading interventions’ most influential aspects as professionals continue to hold key decision-making and governance positions [32–34]. Secondly, studies like Renedo & Marston [18] and Lewis [17] highlights that the way in which professionals view and discuss citizens contains contradictions that maintain the institutional status quo. For example, Lewis [17] discussed how professionals dismissed and undermined engaged service-users of a mental health service, because of their having a mental illness. The professionals called into question the validity of service-users’ contributions by suggesting their mental health issues ‘made them unreasonable’. Renedo & Marston [18] explained that professionals’ contradictory discourse and expectations of engaged citizens limits citizens in the type and scope of contributions they are able to make—e.g. having professional-level skills, while at the same time being a ‘genuine’ citizen in the local area. Such discourses maintain a clear division between a ‘powerful us’ (professionals) and disempowered ‘others’ (engaged citizens). Constraining contextual factors, which may make it harder to address power imbalances, include disadvantaged communities used to being at the lower end of the privilege spectrum, and organisations remaining symbolic institutions of power and hierarchy [9, 17, 32–34]). For example, Lewis [17] showed that a lack of institutional status *(context)* can lead to citizens feeling out of place and unsure of how to contribute to organisationally run meetings *(mechanism)*. This led to some citizens feeling silenced *(outcome)*, while others felt angry and shouted out their views *(outcome)*, which in turn was dismissed by professionals *(outcome)*. Ultimately, CE initiatives will only be successful if organisations address power imbalances, share control with citizens and professionals and citizens view each other as legitimate and equal partners in the interventions [17, 18, 33].

### Local reference panel reflections

The panel recognised the importance of the equal status between organisations and citizens. They felt that achieving such a balance would require open and honest discussions between organisations and citizens about their respective roles in a more equal CE structure.

#### Guiding principle 6: Invest in citizens who feel they lack the skills and confidence to engage

Organisations should offer learning opportunities to citizens who feel they lack the skills and confidence to engage. Without being offered the opportunity to learn the required skills and capabilities, many, more vulnerable, citizens will likely feel unable to effectively engage [10, 12, 16, 18, 32]. For example, Crondahl & Eklund Karlsson [10] evaluated a CE learning intervention, which aimed to empower socially excluded and discriminated against Roma citizens to become health promotion coordinators in their own Roma communities *(context)*. The training programme helped the Roma coordinators to develop a greater sense of control and empowerment *(mechanism)*, which led to increased self-acceptance and to a sense of positive Roma culture *(outcome)* [10]. De Freitas & Martin’s study [16] of a mental health provider’s advocacy project supporting migrants with mental health issues showed how culturally sensitive training programmes empowered disadvantaged service-users. The organisation recognised that the participants did not feel as if they had the required skills and delivered training aimed at raising their awareness about the causes of their disadvantage and the tools they could use to alleviate the causes. Additionally, the already engaged service-users were trained to deliver peer-support groups to other service-users. The direct peer recruitment enabled the marginalised service-users to recognise their own entitlement to participation and enable them to successfully recruit other migrants with mental health issues into the service. The peer supporters themselves increased their social interactions, improved their communication skills and adhered more to their own treatment plans [16]. Constraining contextual factors include organisations that maintain power imbalances, have unclear remits for citizens, and have tense relationships with communities. Organisations operating within such contexts will struggle to provide the right learning opportunities to citizens who do not already feel empowered; again highlighting the importance of first addressing such constraining contextual factors.

### Local reference panel reflections

The panel agreed that in their experience it was difficult to engage citizens who are not already empowered. Most of their citizen-participants not only live in the area, but also work in the local healthcare sector. The stakeholders are still searching for the best ways of engaging more disadvantaged citizens, but suggested ‘buddying up’ vulnerable citizens with the already engaged citizens.

#### Guiding principle 7: Create quick and tangible wins

Quick wins are important for CE interventions to build and maintain momentum among citizens [11, 32, 34, 40, 41]. Hamamoto et al. [11] described how a local community health centre engaged thousands of citizens in tangible projects promoting active living in the area, e.g. a mother’s walking group, bicycle repair and distribution programme. Though the local community had pressing and visible socio-economic needs including a deteriorating infrastructure not easily lending itself to physical activity *(context)*, the early successes in the initial stages of the intervention provided momentum and energy for citizens to come together towards other common and achievable goals *(mechanism)*. This led to thousands of citizens to volunteer for health-related activities and youth programmes *(outcome)*. However, the community centre struggled to engage citizens in broader policy development *(outcome)*, partly because the Centre did not have enough supportive resources or clearly defined policy issues to mobilize the community around. Kegler et al. [41] evaluated communities’ participation in California Healthy Cities and Communities programmes and found that sites, which focused on tangible mobilisation efforts such as neighbourhood clean-ups typically, generated more spin-off activities, and had more citizens participating in projects’ implementation phases. Studies like Durey et al. [32] and Yoo et al. [40] suggest that quick wins are especially important for interventions where communities’ previous experiences of CE, or health and care services more generally, have been negative and failed to show any benefits to citizens*,* this is especially true for CE interventions with marginalised and low-income communities. In such contexts, a lack of quick, concrete improvements can worsen citizens’ feelings of powerlessness and will likely result in citizens being less likely to participate in future interventions [40]. While, quick wins which result in changes that improve services, help to create communities’ trust in the engagement processes and can trigger citizens’ dedication and ability to push through difficulties and obstacles [32, 34].

### Local reference panel reflections

The panel echoed Hamamoto et al.’s [11] findings and highlighted the difficulties in maintaining citizens’ engagement in interventions that had achieved the quick wins and were running smoothly. The panel mentioned that regularly relating to citizens how their input is being used and how it contributes to successful outcomes can be helpful in maintaining citizens’ interest. They felt that such transparency might also force organisations to actually use citizens’ input.

#### Guiding principle 8: Take into account both citizens’ and organisations’ motivations

Organisations should enable citizens to participate in activities and projects that truly interest and motivate them, instead of channelling their participation to other projects [11, 12, 16, 17, 36, 38, 39, 42]. The community centre evaluated by Hamamoto et al. [11], for example, enabled citizens to solely provide input into those projects, which truly interested them, which meant that citizens remained engaged for the entire length of their specific project. As Van Eijk & Steen [38] argued, citizens cannot pay attention to every topic and are often engaged in an ad hoc manner, contingent on specific problems. For example, their study of a mental healthcare provider’s Client Council showed that citizens’ motivations for joining the Council were mostly personal—e.g. because they were a service-user or the carer of a service-user and wanted more social interactions with others in a similar situation. The implication was that as soon as that personal connection disappeared, their commitment to the Council decreased. In Lewis’ [17] study concerning a policy and planning committee operating within a hierarchical organisation *(context)*, service-users’ own negative mental health service-usage experiences motivated them to take part in the committee with the aim of improving mental health services and to find solidarity with others *(mechanism).* However, because the committee was ineffective in addressing the poor quality standards which were the cause of service-users’ negative experiences, the citizens felt they would be better off forming their own forms of active citizenship relating to mental health services *(mechanism)*. Eventually the service-users did split off from the committee and set up, for example, a mental health charity and a mental health social firm *(outcome)* [17]. Such examples show that organisations should be transparent about the problems the organisation is facing and about their own motivations, especially if it is their intention to make cost-savings, and listen openly to citizens’ negative experiences. Aligning motivations can enhance personal citizens’ personal connections with services and can enable longer-term collaboration between citizens and organisations.

### Local reference panel reflections

The panel recognised the importance of aligning CE interventions with citizens’ own interests and motivations. In their experience, for example, citizens are less interested in CE initiatives focusing on an entire municipality; while initiatives centred on their local neighbourhood, attract more input from citizens.

## Discussion

As far as the authors are aware, this is the first review to develop guiding principles for the successful implementation of community engagement interventions. Using the realist methodology, the rapid review identified eight guiding principles and highlighted the different enabling and constraining contextual factors and mechanisms, which influence the effectiveness of CE interventions. The literature findings, which resonated with the Dutch local reference panel, provide policymakers and practice leaders with an understanding of the key principles, which promote the engagement of citizens in the health and care setting. The aim of this information is to enable professionals to implement their own effective CE interventions.

While this review has not examined the interactions between the eight guiding principles, they appear interrelated. For example, those in leadership positions play an important role in ensuring CE interventions are enacted in a safe and trusting environment for citizens, which in turn seems tied into the power imbalances between citizens and organisations. Future studies could examine the nature and extent of the principles’ interactions and how these can be used to reach more ‘meaningful participation’—for example by investigating ‘ripple effect mechanisms’ [43].

Even without the examination of principles’ interrelatedness, it is clear that the existence of power imbalances and organisations’ willingness, or not, to address such imbalances, is an especially important thread throughout the principles. The literature suggests that ‘meaningful participation’ of citizens can only be achieved if organisational processes are adapted to ensure they are inclusive, accessible and supportive of citizens, for example by placing citizens in decision-making and leadership positions and providing relevant learning opportunities [16, 32–34]. This holds especially true for interventions seeking to engage communities with lower levels of capacity and higher levels of deprivation. In such cases, organisations should first invest significant time and resources in developing positive and trusting relationships with communities [10, 16, 40, 42]. However, the literature contains more examples of how failing to build more equal organisational structures results in worsening relationships and the deterioration of citizens’ empowerment. Studies like Carlisle (2010), Lewis [17] and Renedo & Marston [18] have shown that even though organisations implement CE interventions, ostensibly with the aim of involving citizens more deeply in their organisation, professionals continue to maintain their ‘business as usual’ approach.

Future studies will be needed to continue broadening our understanding of CE. Firstly, it remains unclear why professionals and organisations implement CE interventions, but then ‘maintain their business as usual’ approach. Renedo & Marston [18] suggest part of the problem lies in the professional discourse around citizen engagement, but new evaluations could question wider aspects of this problem by investigating other underlying mechanisms and contextual factors, which prevent organisations from fully adapting their processes and structures. As CE is expected to bring a wider range of services together specifically around citizens’ views and needs, new studies could investigate, for example, how service fragmentation and funding competition hamper professionals’ willingness to truly take on board citizens’ more holistic and potentially remit-transcending views. Some of the studies included in this review indicate that service fragmentation and a lack of funding aggravate uncollaborative citizen-professional relationships, especially if professionals place an emphasis on the self-sustainability of (marginalised) communities [31, 9, 33]. Secondly, while there have been some studies highlighting how CE interventions which address power imbalances can tailor specific health and care services or local neighbourhoods to citizens’ needs [32–34, 39]; little is known about whether CE actually enables the implementation of new collaborative models of care centred on citizens’ preferences. Finally, too few evaluations have investigated interventions involving low-income or ethnically diverse communities. There are even fewer studies focusing on other vulnerable or disadvantaged groups like the frail elderly, LGBTQ citizens, or less abled citizens [13, 44–46]. This could partly be because not many CE interventions with such target groups have been implemented, in which case new studies could examine why that is the case—perhaps the reason lies partly in the context of the disadvantage and marginalisation such groups face. Because contexts of power imbalances, marginalisation and discrimination are hugely influential, such studies will be key to ensuring our understanding of CE is more inclusive and complete and can be tailored more closely to different citizens’ needs. As the local reference panel pointed out, citizens are not one homogenous group with the same needs, priorities and preferences.

Studies using a wider-range of quantitative methodologies and those reporting on the more negative results or aspects of the studied CE interventions would help close such gaps. To date, most CE evaluations have been qualitative and based on case studies and have not explicitly discussed the studies’ negative results. These case studies have provided rich anecdotal evidence, but to further develop our understanding of which CE interventions work or not, for whom, how, in which contexts and to what extent, new studies should use mixed-methods in order to quantify findings, thus providing a richer evidence-base. The authors will attempt to address such remaining gaps in the multiple-case study going forward, using the principles and the underlying CMOs as the initial programme theories.

On a separate note, though the focus of this paper was not the application of the realist methodology, important questions arose during the review’s data analysis stage. The first issue relates to theory development using CMO configurations. While most realist papers clearly highlight that CMO configurations were key to the development of the theories under discussion, most do not actually describe how the CMO configurations then led to those theories [47]. Within the papers that do describe this analytical process, there seems to be no consistency as to whether the theories are centred on the contexts, mechanisms, or outcomes of the configurations. For example, previous evaluators have put interventions as related to outcomes central [21, 23], others have placed only outcomes in the limelight (e.g. [48]) or outcomes and contexts [49], and similarly to Kane et al. [50], we saw mechanisms as critical for our guiding principles. The methodology’s inherent flexibility brings many, creative, benefits, however, it also raises important questions regarding the generation of results. For example, it is currently not clear whether we would have drawn the same conclusions if we had chosen context or outcomes as the core of this review’s analysis. For example, due to the review’s focus on mechanisms—i.e. what makes citizens or communities want to participate or not—and our aim of providing policymakers and professionals with the evidence to implement their own effective CE strategies—the outcomes within our individual CMO configurations often relate to citizens’ or communities’ behavioural changes and the impact on organisational processes, rather than say the impact on citizens’ health and wellbeing. However, the local reference panel’s valuable input indicates that our results have face validity.

Relatedly, the methodology’s flexibility and dynamic nature is again one of its key strengths as it provides rich and detailed information, partly because of its recognition that interventions and their contexts are complex and varying. However, there is a tension between the recognition that all contexts are in a way unique, and the generalisability of the results. This tension is only partly addressed by searching for the same mechanisms and outcomes in different contexts. Ultimately, if the methodology is to continue to evolve and improve, realist evaluators should not only be transparent about how they constructed CMOs and generated theories, but also why they choose that specific approach and endeavour to show that the results are indeed generalisable across different contexts and care settings.

### Study limitations

This study has two main limitations. Firstly, though this rapid review’s literature search was systematic, it was by no means exhaustive in a conscious effort to speed up the process and to share the findings as quickly as possible with stakeholders. This limitation has been mitigated by collaborating with the local reference panel to confirm and supplement the findings. Secondly, while the realist methodology is helpful in uncovering multifaceted and complex issues like power imbalances in CE, the methodology is still developing, which means that key concepts are not always understood or applied in the same manner. Other researchers may therefore find it difficult to build on this review’s findings, however, the authors have attempted to address this limitation by clearly stipulating the applied understandings of key concepts and describing, in detail, how and why the CMOs and principles were developed.

## Conclusions

By highlighting the contextual factors and mechanisms, which can influence the outcome of CE interventions, the eight guiding principles can hopefully guide professionals to develop their own successful interventions. While the principles are based on a wide range of contextual factors, professionals are encouraged to interpret and adapt the findings to the contexts of their own local settings and explore which activities and mechanisms would lead to the most inclusive and diverse CE interventions. Organisations should pay specific attention to sources of contextual power imbalances and find the most appropriate ways to empower, motivate and upskill citizens so they may take shared control of initiatives.

## Additional file

Additional file 1: Search strings. (DOCX 32 kb)

## Abbreviations

CE: Community engagement
CMOs: Context-mechanism-outcome configurations
Principles: Action-oriented guiding principles
