# Supplementary material for: Achieving successful community engagement: a rapid realist review
Source: BMC Health Serv Res. 2018 Apr 13;18:285. doi: 10.1186/s12913-018-3090-1 (PMC5899371; doi:10.1186/s12913-018-3090-1)
Supplement: Supplementary file 1 — Search strings. (DOCX 32 kb) [file 12913_2018_3090_MOESM1_ESM.docx]

**Appendix 1: Search strings**

**Embase search strings 1a: 21 February 2017**

| #1 | 'community engagement':ti,ab OR 'community participation':ti,ab OR 'community empowerment':ti,ab OR 'community networks':ti,ab OR 'grassroots participation':ti,ab OR 'grassroots networks':ti,ab OR 'social engagement':ti,ab OR 'community collaboration':ti,ab OR 'citizen engagement':ti,ab OR 'citizen participation':ti,ab OR 'participatory approaches':ti,ab OR 'community engagement strateg*':ti,ab OR 'citizen engagement strateg*':ti,ab OR 'public representation':ti,ab OR 'public engagement':ti,ab OR 'public participation':ti,ab OR 'community representation':ti,ab OR 'citizen representation':ti,ab OR 'cultural representation':ti |
| --- | --- |
| #2 | 'community'/de AND 'empowerment'/de OR 'participatory management'/de |
| #3 | #1 or #2 |
| #4 | health:ti,ab OR 'public health':ti,ab OR 'health promotion':ti,ab OR 'population health':ti,ab OR 'health care policy':ti,ab OR 'health care planning':ti,ab OR 'public health planning':ti,ab OR 'health care design':ti,ab OR 'health care governance':ti,ab OR 'health care decision-making':ti,ab OR governance:ti |
| #5 | 'health'/de OR 'public health'/de OR 'health promotion'/de OR 'health care policy'/de OR 'health care planning'/de OR ('health care'/de AND 'decision making'/de) |
| #6 | 'social care':ti,ab OR 'social care policy':ti,ab OR 'social care planning':ti,ab OR 'social care design':ti,ab OR 'social care governance':ti,ab OR 'social care decision-making':ti,ab |
| #7 | 'social care'/de OR ('social care'/de AND ('decision making'/de OR 'planning'/de)) |
| #8 | 'hiap' OR 'health in all polic*' OR 'intersectoral collaboration' OR 'intersectoral action' OR 'intersectoral approaches' OR ('intersectoral action' NEAR/3 health AND equity) OR 'social determinants' NEAR/3 health OR 'social determinants of health'/de |
| #9 | ((quality OR sustainability OR satisfaction OR trust) NEAR/3 ('health care' OR 'health services' OR 'social care' OR 'social services' OR care)):ti,ab OR (system* NEAR/3 sustainability):ti,ab OR (system* NEAR/3 integration):ti,ab OR 'health care quality'/de |
| #10 | #3 AND (#4 OR #5 OR #6 OR #7 OR #8 OR #9) |
| #11 | 'disadvantaged population*':ti,ab OR 'disadvantaged groups':ti,ab OR 'disadvantaged neighbourhoods':ti,ab OR 'marginalised population*':ti,ab OR 'marginalised groups':ti,ab OR 'marginalised neighbourhoods':ti,ab OR 'socially excluded populations':ti,ab OR 'socially excluded groups':ti,ab OR 'vulnerable population*':ti,ab OR 'vulnerable groups':ti,ab OR 'frail elderly':ti,ab OR 'ethnic minorit*':ti,ab OR 'ethnic communities':ti,ab OR 'ethnic groups':ti,ab OR 'ethnic neighbourhoods':ti,ab OR refugees:ti,ab OR migrants:ti,ab OR 'disabled persons':ti,ab OR 'sexual minorit*':ti,ab OR lgbt:ti,ab OR 'lower educated':ti,ab OR 'socio-econom* disadvantaged group*':ti,ab OR 'low income households':ti,ab OR 'low income families':ti,ab OR 'socioeconomic factors':ti,ab |
| #12 | 'vulnerable population'/mj OR 'ethnic group'/mj OR 'minority group'/mj OR 'frail elderly'/mj OR 'refugee'/mj OR 'migrant'/mj OR 'sexual minority'/mj OR 'lgbt people'/mj OR 'disabled person'/mj OR 'lowest income group'/mj OR 'social status'/mj OR 'social class'/mj OR 'educational status'/mj OR 'socioeconomics'/mj |
| #13 | #10 AND (#11 OR #12) |
| #14 | 'health equit*':ti,ab OR 'health inequit*':ti,ab OR 'health inequalit*':ti,ab OR 'health disparit*':ti,ab OR 'healthcare disparit*':ti,ab OR 'health care disparit*':ti,ab OR 'care disparit*':ti,ab OR 'health determinants':ti,ab OR (determinants NEAR/3 health):ti,ab OR 'socio-economic factors':ti,ab OR 'social exclusion':ti,ab OR ('health services' NEAR/3 accessibility):ti,ab OR 'health status disparit*':ti,ab OR 'burden of disease':ti,ab OR inequality:ti,ab OR inequalities:ti,ab OR inequity:ti,ab OR inequities:ti,ab OR gaps:ti,ab |
| #15 | 'health equity'/mj OR 'health disparity'/mj OR 'health care disparity'/mj OR 'health status'/mj OR 'social determinants of health'/mj OR 'socioeconomics'/mj OR 'social exclusion'/mj OR 'health care access'/mj |
| #16 | #10 AND (#14 OR #15) |
| #17 | 'health outcome*':ti,ab OR 'health literacy':ti,ab OR 'health behavior':ti,ab OR 'health behaviour':ti,ab OR 'quality of life':ti OR 'physical functioning':ti,ab OR 'mental functioning':ti,ab OR 'self-management':ti,ab OR activation:ti,ab OR 'self efficacy':ti,ab |
| #18 | 'treatment outcome'/mj OR 'health literacy'/mj OR 'health behavior'/mj OR 'quality of life'/mj OR 'self care'/mj OR 'self actualization'/mj |
| #19 | #10 AND (#17 OR #18) |
| #20 | 'wellbeing outcome*':ti,ab OR 'emotional wellbeing':ti,ab OR 'mental wellbeing':ti,ab OR 'well-being outcome*':ti,ab OR 'emotional well-being':ti,ab OR 'mental well-being':ti,ab OR 'social inclusion':ti,ab OR 'voice and agency':ti,ab OR 'social capital':ti,ab OR 'community capacity building':ti,ab OR 'sense of control':ti,ab OR (sense NEAR/4 integration):ti,ab OR 'social cohesion':ti,ab OR 'active citizenship':ti,ab OR 'independence':ti,ab |
| #21 | 'wellbeing'/mj OR 'social exclusion'/mj OR 'social capital'/mj OR ('community'/de AND 'capacity building'/de) OR 'integration'/mj OR 'citizenship'/mj OR 'independence'/mj |
| #22 | #10 AND (#20 OR #21) |
| #23 | #13 OR #16 OR #19 OR #22 |
| #24 | #23 AND (english:la OR dutch:la) |
| #25 | letter:it OR note:it OR erratum:it OR news:it OR 'conference abstract':it OR 'conference paper':it OR 'conference review':it |
| #26 | #24 NOT #25 |
| #27 | engagement:ti OR participat*:ti OR empower*:ti OR network*:ti OR communit*:ti OR citizen*:ti OR transdisciplin*:ti OR intersectoral:ti OR involvement:ti OR 'social determinant*':ti OR 'social capital':ti OR disadvant*:ti OR disparat*:ti OR cultural:ti OR polic*:ti OR organizational:ti OR project*:ti OR program*:ti OR care:ti OR healthcare:ti OR 'public health':ti OR 'population health':ti OR services:ti OR 'health outcome*':ti OR 'health equity':ti OR inequal*:ti OR inequit*:ti |
| #28 | #26 AND #27 |

**Embase search strings 1b: 21 February 2017**

| #1 | 'community engagement':ti,ab OR 'community participation':ti,ab OR 'community empowerment':ti,ab OR 'community networks':ti,ab OR 'grassroots participation':ti,ab OR 'grassroots networks':ti,ab OR 'social engagement':ti,ab OR 'community collaboration':ti,ab OR 'citizen engagement':ti,ab OR 'citizen participation':ti,ab OR 'participatory approaches':ti,ab OR 'community engagement strateg*':ti,ab OR 'citizen engagement strateg*':ti,ab OR 'public representation':ti,ab OR 'public engagement':ti,ab OR 'public participation':ti,ab OR 'community representation':ti,ab OR 'citizen representation':ti,ab OR 'cultural representation':ti,ab OR 'cultural sensitivity':ti,ab OR 'citizen science':ti,ab |
| --- | --- |
| #2 | 'community'/de AND 'empowerment'/de OR 'participatory management'/de |
| #3 | #1 OR #2 |
| #4 | health:ti,ab OR 'public health':ti,ab OR 'health promotion':ti,ab OR 'population health':ti,ab OR 'health care policy':ti,ab OR 'health care planning':ti,ab OR 'public health planning':ti,ab OR 'health care design':ti,ab OR 'health care governance':ti,ab OR 'health care decision-making':ti,ab OR governance:ti |
| #5 | 'health'/de OR 'public health'/de OR 'health promotion'/de OR 'health care policy'/de OR 'health care planning'/de OR ('health care'/de AND 'decision making'/de) |
| #6 | 'social care':ti,ab OR 'social care policy':ti,ab OR 'social care planning':ti,ab OR 'social care design':ti,ab OR 'social care governance':ti,ab OR 'social care decision-making':ti,ab |
| #7 | 'social care'/de OR ('social care'/de AND ('decision making'/de OR 'planning'/de)) |
| #8 | 'hiap' OR 'health in all polic*' OR 'intersectoral collaboration' OR 'intersectoral action' OR 'intersectoral approaches' OR ('intersectoral action' NEAR/3 health AND equity) OR 'social determinants' NEAR/3 health OR 'social determinants of health'/de |
| #9 | ((quality OR sustainability OR satisfaction OR trust) NEAR/3 ('health care' OR 'health services' OR 'social care' OR 'social services' OR care)):ti,ab OR (system* NEAR/3 sustainability):ti,ab OR (system* NEAR/3 integration):ti,ab OR 'health care quality'/de |
| #10 | #3 AND (#4 OR #5 OR #6 OR #7 OR #8 OR #9) |
| #11 | principle*:ti OR theory:ti OR theories:ti OR theoretical:ti OR mechanism*:ti OR method*:ti OR model:ti OR models:ti OR measuring:ti OR practice*:ti OR approach*:ti OR strateg*:ti OR process*:ti OR program:ti AND development:ti OR design:ti OR evidence:ti OR evaluation:ti OR insight*:ti OR ingredient*:ti OR key:ti OR context*:ti OR role:ti |
| #12 | #10 AND #11 |
| #13 | engagement:ti OR participat*:ti OR empower*:ti OR network*:ti OR communit*:ti OR citizen*:ti OR transdisciplin*:ti OR intersectoral:ti OR involvement:ti OR 'social determinant*':ti OR 'social capital':ti OR disadvant*:ti OR disparat*:ti OR cultural:ti OR polic*:ti OR organizational:ti OR project*:ti OR program*:ti OR care:ti OR healthcare:ti OR 'public health':ti OR 'population health':ti OR services:ti OR 'health outcome*':ti OR 'health equity':ti OR inequal*:ti OR inequit*:ti |
| #14 | #12 AND #13 |
| #15 | #14 AND (english:la OR dutch:la) |
| #16 | letter:it OR note:it OR erratum:it OR news:it OR 'conference abstract':it OR 'conference paper':it OR 'conference review':it |
| #17 | #15 NOT #16 |

**Scopus search strings 1a: 2 March 2017**

( ( TITLE-ABS ( ( community-engagement )  OR  ( community-participation )  OR  ( community-empowerment )  OR  ( community-networks )  OR  ( grassroots-participation )  OR  ( grassroots-networks )  OR  ( social-engagement )  OR  ( community-collaboration )  OR  ( citizen-engagement )  OR  ( citizen-participation )  OR  ( participatory-approaches )  OR  ( community-engagement-strateg* )  OR  ( citizen-engagement-strateg* )  OR  ( public-representation )  OR  ( public-engagement )  OR  ( public-participation )  OR  ( community-representation )  OR  ( citizen-representation )  OR  ( cultural-representation )  OR  ( cultural-sensitivity )  OR  ( citizen-science ) )  AND  ( ( TITLE-ABS ( health  OR  ( public-health )  OR  ( health-promotion )  OR  ( population-health )  OR  ( health-care-policy )  OR  ( health-care-planning )  OR  ( public-health-planning )  OR  ( health-care-design )  OR  ( health-care-governance )  OR  ( health-care-decision-making ) )  OR  TITLE ( governance ) )  OR  ( TITLE-ABS ( ( social-care )  OR  ( social-care-policy )  OR  ( social-care-planning )  OR  ( social-care-design )  OR  ( social-care-governance )  OR  ( social-care-decision-making ) ) )  OR  ( TITLE-ABS ( hiap  OR  ( health-in-all  AND polic* )  OR  ( intersectoral-collaboration )  OR  ( intersectoral-action )  OR  ( intersectoral-approaches )  OR  ( ( social-determinants )  W/3  health ) ) )  OR  ( TITLE-ABS ( ( ( quality  OR  sustainability  OR  satisfaction  OR  trust )  W/3  ( ( health-care )  OR  ( health-services )  OR  ( social-care )  OR  ( social-services )  OR  care ) )  OR  ( system*  W/3  sustainability )  OR  ( system*  W/3  integration ) ) ) ) )  AND  ( ( TITLE-ABS ( ( disadvantaged-population* )  OR  ( disadvantaged-groups )  OR  ( disadvantaged  AND neighbourhoods )  OR  ( marginalised-population* )  OR  ( marginalised-groups )  OR  ( marginalised-neighbourhoods )  OR  ( socially-excluded-populations )  OR  ( socially-excluded  AND groups )  OR  ( vulnerable-population* )  OR  ( vulnerable-groups )  OR  ( frail-elderly )  OR  ( ethnic-minorit* )  OR  ( ethnic-communities )  OR  ( ethnic-groups )  OR  ( ethnic-neighbourhoods )  OR  refugees  OR  migrants  OR  ( disabled-persons )  OR  ( sexual  AND minorit* )  OR  lgbt  OR  ( lower-educated )  OR  ( socio-econom*-disadvantaged-group* )  OR  ( socio-econom*-disadvantaged-group* )  OR  ( low-income-households )  OR  ( low-income-families )  OR  ( socioeconomic-factors ) )  OR  TITLE-ABS ( ( health-equit* )  OR  ( health-inequit* )  OR  ( health-inequalit* )  OR  ( health-disparit* )  OR  ( healthcare-disparit* )  OR  ( health-care-disparit* )  OR  ( care-disparit* )  OR  ( health-determinants )  OR  ( determinants  W/3  health )  OR  ( socio-economic-factors )  OR  ( social  AND exclusion )  OR  ( ( health-services )  W/3  accessibility )  OR  ( health-status-disparit* )  OR  ( burden-of-disease )  OR  inequality  OR  inequalities  OR  inequity  OR  inequities  OR  gaps )  OR  TITLE-ABS ( ( health-outcome* )  OR  ( health-literacy )  OR  ( health-behavior )  OR  ( health-behaviour )  OR  ( quality-of-life )  OR  ( physical-functioning )  OR  ( mental-functioning )  OR  ( self-management )  OR  activation  OR  ( self-efficacy ) )  OR  TITLE-ABS ( ( wellbeing-outcome* )  OR  ( emotional-wellbeing )  OR  ( mental-wellbeing )  OR  ( well-being-outcome* )  OR  ( emotional-well-being )  OR  ( mental-well-being )  OR  ( social-inclusion )  OR  ( social-capital )  OR  ( community-capacity  AND building )  OR  ( sense-of-control )  OR  ( sense  W/4  integration )  OR  ( social-cohesion )  OR  ( active-citizenship )  OR  independence ) ) ) )  AND  ( TITLE ( engagement  OR  participat*  OR  empower*  OR  network*  OR  communit*  OR  citizen*  OR  transdisciplin*  OR  intersectoral  OR  involvement  OR  ( social-determinant* )  OR  ( social-capital )  OR  disadvant*  OR  disparat*  OR  cultural  OR  polic*  OR  organizational  OR  project*  OR  program*  OR  care  OR  healthcare  OR  ( public-health )  OR  ( population-health )  OR  services  OR  ( health-outcome* )  OR  ( health-equity )  OR  inequal*  OR  inequit* )  AND  ( LANGUAGE ( english  OR  dutch )  AND NOT  ( DOCTYPE ( cp )  OR  DOCTYPE ( cr )  OR  DOCTYPE ( le )  OR  DOCTYPE ( no )  OR  DOCTYPE ( er ) ) )

**Scopus search strings 1b: 2 March 2017**

( ( TITLE ( ( community-engagement ) OR ( community-participation ) OR ( community-empowerment ) OR ( community-networks ) OR ( grassroots-participation ) OR ( grassroots-networks ) OR ( social-engagement ) OR ( community-collaboration ) OR ( citizen-engagement ) OR ( citizen-participation ) OR ( participatory-approaches ) OR ( community-engagement-strateg* ) OR ( citizen-engagement-strateg* ) OR ( public-representation ) OR ( public-engagement ) OR ( public-participation ) OR ( community-representation ) OR ( citizen-representation ) OR ( cultural-representation ) OR ( cultural-sensitivity ) OR ( citizen-science ) ) ) AND ( TITLE ( principle* OR theory OR theories OR theoretical OR mechanism* OR method* OR model OR models OR measuring OR practice* OR approach* OR strateg* OR process* OR ( program-development ) OR design OR evidence OR evaluation OR insight* OR ingredient* OR key OR context* OR role ) ) AND ( TITLE ( engagement OR participat* OR empower* OR network* OR communit* OR citizen* OR transdisciplin* OR intersectoral OR involvement OR ( social-determinant* ) OR ( social-capital ) OR disadvant* OR disparat* OR cultural OR polic* OR organizational OR project* OR program* OR care OR healthcare OR ( public-health ) OR ( population-health ) OR services OR ( health-outcome* ) OR ( health-equity ) OR inequal* OR inequit* ) ) AND ( LANGUAGE ( english OR dutch ) AND NOT ( DOCTYPE ( cp ) OR DOCTYPE ( cr ) OR DOCTYPE ( le ) OR DOCTYPE ( no ) OR DOCTYPE ( er ) ) ) ) AND ( ( TITLE-ABS ( health OR ( public-health ) OR ( health-promotion ) OR ( population-health ) OR ( health-care-policy ) OR ( health-care-planning ) OR ( public-health-planning ) OR ( health-care-design ) OR ( health-care-governance ) OR ( health-care-decision-making ) ) OR TITLE ( governance ) ) OR ( TITLE-ABS ( ( social-care ) OR ( social-care-policy ) OR ( social-care-planning ) OR ( social-care-design ) OR ( social-care-governance ) OR ( social-care-decision-making ) ) ) OR ( TITLE-ABS ( hiap OR ( health-in-all AND polic* ) OR ( intersectoral-collaboration ) OR ( intersectoral-action ) OR ( intersectoral-approaches ) OR ( ( social-determinants ) W/3 health ) ) ) OR ( TITLE-ABS ( ( ( quality OR sustainability OR satisfaction OR trust ) W/3 ( ( health-care ) OR ( health-services ) OR ( social-care ) OR ( social-services ) OR care ) ) OR ( system* W/3 sustainability ) OR ( system* W/3 integration ) ) ) OR ( TITLE-ABS ( ( ( quality OR sustainability OR satisfaction OR trust ) W/3 ( ( health-care ) OR ( health-services ) OR ( social-care ) OR ( social-services ) OR care ) ) OR ( system* W/3 sustainability ) OR ( system* W/3 integration ) ) ) )
